# Meniscal pathology - the evidence for treatment

**DOI:** 10.1186/ar4515

**Published:** 2014-03-20

**Authors:** Veronica Mezhov, Andrew J Teichtahl, Rupert Strasser, Anita E Wluka, Flavia M Cicuttini

**Affiliations:** 1 Department of Epidemiology and Preventive Medicine, School of Public Health and Preventive Medicine, Monash University, Alfred Hospital, Melbourne, VIC 3004, Australia

## Abstract

Whilst arthroscopic surgery for the treatment of meniscal tears is the most commonly performed orthopaedic surgery, meniscal tears at the knee are frequently identified on magnetic resonance imaging in adults with and without knee pain. The evidence for arthroscopic treatment of meniscal tears is controversial and lacks a supporting evidence base; it may be no more efficacious than conservative therapies. Surgical approaches to the treatment of meniscal pathology can be broadly categorised into those in which partial menisectomy or repair are performed. This review highlights that the major factor determining the choice of operative approach is age: meniscal repair is performed exclusively on younger populations, while older populations are subject to partial menisectomy procedures. This is probably because the meniscus is less amenable to repair in the older population where other degenerative changes co-exist. In middle-aged to older adults, arthroscopic partial menisectomy (APM) may treat the meniscus tear, but does not address the degenerative whole organ disease of knee osteoarthritis. Thus far, there is no convincing evidence that operative approaches are superior to conservative measures as the first-line treatment of older people with knee pain and meniscal tears. However, in two randomised controlled trials (RCTs) approximately one-third of subjects in the exercise groups had persisting knee pain with some evidence of improvement following APM, although the characteristics of this subgroup are unclear. From the available data, a first-line trial of conservative therapy, which includes weight loss, is recommended for the treatment of degenerative meniscal tears in older adults. The exception to this may be when mechanical symptoms, such as knee locking, predominate. Although requiring corroboration by RCTs, there is accumulating evidence from cohort studies and case series that meniscal repair rather than APM may improve function and reduce the long-term risk of knee osteoarthritis in young adults. There is no clear evidence from RCTs that one surgical method of meniscal repair is superior to another.

## Introduction

The menisci are fibrocartilaginous structures located between the medial and lateral tibiofemoral joints that function to reduce knee joint loads by shock absorption. When severe, meniscal tears are implicated in knee locking and pain. However, using magnetic resonance imaging, meniscal tears are present in approximately 20% of people without knee symptoms [1]. Indeed, meniscal pathology is now considered part of the spectrum of pathology seen in knee osteoarthritis (OA) and meniscal tears are a risk factor for further deleterious structural change, such as accelerated articular cartilage loss [2].

In 1942, McMurray commented that ‘a far too common error is shown in the incomplete removal of the injured meniscus’ [3]. However, total menisectomy has since then been shown detrimental to the knee joint. In 1948, after total menisectomy, anteroposterior ridging occurred at the femoral condyle, with flattening of the articular surface and joint space narrowing [4]. Contemporary studies have substantiated these findings, with radiographic OA being 14 times more common in people two decades after having had a total menisectomy compared with age-matched and gender-matched controls [5]. Recently, a prospective longitudinal 40-year follow-up study examined people who underwent open total menisectomy as adolescents for isolated meniscal pathology. The results showed that total menisectomy increased the risk of symptomatic knee OA in later life, with a resultant 132-fold increase in the rate of knee replacement compared with geographical and age-matched controls [6].

The advent of arthroscopic surgery has enabled the resection of minimum amounts of damaged meniscal tissue, and even meniscal repair. *In vitro* studies have demonstrated that joint stresses are related to the amount of meniscus removed [7]. When patients undergoing partial or total menisectomy have been compared, the amount of tissue resected was demonstrated to be inversely related to knee function [8]. Arthroscopic partial menisectomy (APM) remains the most common surgical intervention for meniscal pathology and the most common orthopaedic surgical procedure in the United States, with more than 465,000 people undergoing the procedure annually [9]. Nevertheless, there is a paucity of data examining the efficacy of treatments available for meniscal tears. For instance, there is no study that has compared whether APM is superior to nonoperative therapy in the treatment of traumatic meniscal tears. There are, however, some commonalities emerging from both randomised controlled trials (RCTs) and observational studies that have examined therapeutic interventions for meniscal pathology.

This review aims to examine the evidence underlying the use of arthroscopic interventions and conservative measures such as physical therapy, weight loss and analgesia in the management of meniscal tears.

## Evidence for arthoscopic partial menisectomy

Several studies have examined the evidence for APM, either as cohort studies or RCTs (Table 1). Other than one study [10], all data examining APM have examined subjects with a mean age of 43 years or older. These studies have failed to demonstrate superiority of APM over conservative measures.

**Table 1:** Evidence for arthroscopic partial meniscectomy

**Study**	**Study description**	**Group 1**	**Group 2**	**Outcome measure**	**Results**	**Conclusion**
**Randomised control trials**						
Sihoven and colleagues, 2013 [18]	Multicentre with symptomatic medial meniscal tear	APM	Sham surgery	Symptoms	No significant between-group differences from baseline to 12 months in any primary outcome (LKS, WOMET and knee pain after exercise)	APM not superior to sham surgery in reducing knee symptoms at 12 months
Yim and colleagues, 2013 [16]	Degenerative horizontal tear of posterior horn of medial meniscus on MRI; mean age 53.8 years (range 43 to 62 years); follow-up 2 years	APM, *n* = 50	Strengthening exercises, *n* = 52	Symptoms	Both groups reported an improvement in knee pain, function and a high level of treatment satisfaction using VAS, LKS, Tegner activity scale, patient subjective knee pain and satisfaction. No significant between-group differences	APM not superior to strengthening exercises in terms of improved knee pain, function or treatment satisfaction
Katz and colleagues, 2013 [15]	Symptomatic meniscal tear; age ≥45 years; 6-month and 12-month follow-up	APM and postoperative PT, *n* = 161; mean age 59.9 ± 7.9	PT alone, *n* = 169; mean age 57.8 ± 6.8	Symptoms	WOMAC at 6 and 12 months improvement in both groups but no between-group differences; 30% crossover from PT alone within first 6 months	APM + PT not superior to PT for pain reduction
Herrlin and colleagues, 2013 [17]	Symptomatic medial meniscal tear and radiographic OA; 24-month and 60-month follow-up	APM followed by exercise therapy for 2 months, *n* = 47; median age 54 years	Exercise alone, *n* = 49; median age 56 years	Symptoms	Clinical improvement in both groups on all subscales of KOOS, LKS and VAS (*P* <0.0001). One-third of exercise-alone patients that failed to respond had a benefit from then having APM	APM + exercise not superior to exercise alone
Herrlin and colleagues, 2007 [14]	Knee pain and underlying OA with medial meniscal tear; mean age 56 years; 8-week and 6-month follow-up	APM and supervised exercise, *n* = 47	Supervised exercise alone, *n* = 43	Symptoms	Both groups reported decreased knee pain, improved function and high satisfaction. No between-group differences	APM + exercise not superior to exercise alone
Beidert, 2000 [10]	Painful intrasubstance medial meniscal tear; mean age 30.4 years (range 16 to 50 years); 26.5-month follow-up	Group D: APM, *n =* 11	Group A: PT and NSAIDs, *n =* 12	Symptoms	Normal/near-normal IKDC. Group A. 75%; Group D. 100%, *P* = 0.006	APM superior to conservative therapy
**Cohort studies**						
Englund and Lohmander, 2004 [11]	Retrospective case–control study; meniscal Resection 15 to 22 years prior; mean age 54 years at follow-up (±11 years)	APM or total menisectomy, *n =* 317	Control group with no meniscal tear, previous surgery or cruciate pathology, *n* =68	Structure	Radiographic (RR 5.4, 95% CI 2.5 to 13) and symptomatic (RR 2.6, 95% CI 1.3 to 6.1) knee OA more common in operated knees than in controls. Total meniscectomy rather than APM had higher likelihood of knee OA (OR 3.6, 95% CI 1.4 to 9.4)	Menisectomy associated with higher risk of developing knee OA. APM associated with less radiographic knee OA than total menisectomy
Englund and colleagues, 2003 [12]	Retrospective analyses of patients who had undergone menisectomy in an orthopaedic hospital 16 years earlier; mean age 54 years at follow-up (±12 years)	APM or subtotal menisectomy, *n* = 155; mean age 54.3 years	Age, gender and BMI matched controls, *n* = 68; mean age: 56.3 years	Structure	Increased RR of knee OA (RR 4.8, 95% CI 2.2 to 12) and symptom development (RR 2.6, 95% CI 1.6 to 4.7) of knee OA in meniscectomy group. Subtotal menisectomy associated with significantly worse joint space narrowing and KOOS scores than APM	APM or subtotal associated with high risk of radiographic and symptomatic OA at 16-year follow-up. Outcomes worse in degenerative tears and extensive resection

APM, arthroscopic partial menisectomy; BMI, body mass index; CI, confidence interval; MRI, magnetic resonance imaging; NSAID, nonsteroidal anti-inflammatory drug; OA, osteoarthritis; PT, physical therapy; RR, relative risk. Western Ontario Meniscal Evaluation Tool (WOMET) is a disease-specific quality-of-life measurement tool for patients with meniscal lesions looking at symptoms (pain, giving way, swelling, stiffness, numbness, loss of motion), sports/recreation/lifestyle/work and emotion. Western Ontario and McMaster Universities Arthritis Index (WOMAC) evaluates the condition of patients with osteoarthritis of the knee and hip, including pain, stiffness, and physical functioning of the joints. Knee Injury and Osteoarthritis Outcome Score (KOOS) evaluates short-term and long-term patient-related outcomes following injury including pain, other symptoms such as catching/locking/swelling, activities of daily living, sport and recreation function, and knee-related quality of life. Lysholm knee scoring (LKS) scale for knee ligament injuries including pain, swelling, locking, limping, stair climbing, support and squatting. Visual analogue scale (VAS) is a subjective measurement of pain consisting of a line 10 cm long where on one end is ‘no pain’ and on the other is the ‘worst pain imaginable’. International Knee Documentation Committee score (IKDC) is a score to evaluate knee ligament injuries including three domains of symptoms (pain, locking, catching, swelling, stiffness), sports and daily activities and current knee function (compared with old knee function).

### Nonrandomised studies

Three nonrandomised studies have examined the efficacy of menisectomy [11-13]. As opposed to RCTs, which have primarily focused on pain as the primary outcome, cohort studies have focused on the development of incident radiographic knee OA as the outcome of interest. A study examined the risk of developing knee OA following either partial, subtotal or total menisectomy and compared the risk against people who had a meniscal tear but did not undergo menisectomy 15 to 22 years prior [11]. This study found that partial menisectomy was associated with less radiographic OA than was total menisectomy. Nevertheless, symptomatic radiographic knee OA was more likely to occur in operated knees (27%) than in control knees (10%) (relative risk = 2.6; 95% confidence interval = 1.3 to 6.1), regardless of the type of resection performed [11].

In a retrospective study that compared outcomes of people with intact anterior cruciate ligaments (ACLs) who had undergone APM for an isolated meniscal tear, it was found that there was a high risk of radiographic and symptomatic OA at 16-year follow-up [12]. In subgroup analyses, outcomes were worse in those with degenerative tears and extensive resection. Such findings prompted the authors to conclude that degenerative meniscal tears may be associated with incipient OA, and that the meniscal tear may herald the onset of disease.

The final cohort study examined older people with symptomatic meniscal tears, although the symptoms were ill-defined [13]. Participants underwent debridement of unstable articular cartilage and were subject to either arthroscopic washout or APM. At 12 months, there was greater improvement in symptoms after APM than washout alone, with lower analgesic requirements. However, these results should be taken in the context that most participants underwent APM (*n* = 126), with only 13 subjects having had a washout. Such a disparity in numbers between the groups challenges the results of the study and the conclusion that APM assists in symptom control.

### Randomised controlled trials

Data from RCTs have yielded conflicting results regarding the efficacy of APM versus conservative therapies (Table 1). As opposed to cohort studies, which have primarily focused on incident radiographic knee OA as the primary outcome, RCTs have predominantly focused on pain as the outcome of interest.

Four RCTs have compared APM with physical therapy in older (mean age ≥45 years) individuals with symptomatic knee OA [14-17], while one RCT compared APM with sham surgery in people with medial mensical tears [18]. The primary outcomes in these studies were clinical measures of pain and function, using validated instruments, including the Western Ontario and McMaster Universities Arthritic Index, the Knee Injury and Osteroarthritis Outcome Score, the Lysholm Knee Score, the Tegner activity level and visual analogue scales. In all RCTs, both the APM and physical therapy groups showed clinical improvements from baseline to follow-up, although superiority of APM compared with physical therapy could not be demonstrated at any time point. In the most recent RCT [16], no significant differences in terms of relief of knee pain, improved knee function or patient satisfaction between APM and strengthening exercises could be discerned over 2 years of follow-up. In the study by Herrlin and colleagues, one-third of the patients in the exercise group had persisting and disabling knee symptoms after exercise therapy, but improved to the same degree as the APM group when APM was then employed among people who had initially failed to respond to exercise [17]. Nevertheless, the group who required eventual APM were ill-defined. Possibly their symptoms were of a major mechanical origin, whereby knee locking predominated, and such a select subgroup may therefore benefit from APM as first-line treatment. One must acknowledge, however, that two-thirds of subjects responded to exercise therapy, avoiding APM. Caution should therefore be exercised when advocating APM over conservative therapies from this study. No between-group differences existed for improvement in pain levels 12 months postprocedure for both the APM and sham surgery groups in people with medial meniscal tears [18].

Another small RCT examined people with a mean age of 30.4 years (range 16 to 50 years) with a symptomatic medial meniscal tear and randomised them to several treatment arms, which included conservative treatment (nonsteroidal anti-inflammatory drugs and local physical therapy, *n* = 12) and APM (*n* = 11) [10]. At just over 2-year follow-up, people who had an APM demonstrated better outcomes than conservative therapies. Indeed all subjects had a near-normal International Knee Documentation Committee (IKDC) score with APM, compared with 75% with conservative measures (*P* = 0.006). The IKDC is a composite score that evaluates ligament injuries examining four major domains of symptoms (pain, locking, swelling, stiffness). Such results may therefore support the use of APM among younger people with meniscal tears. However, this study was limited by the small number of subjects completing the RCT (*n* = 23), and these results need to be corroborated in a larger RCT. It is also unclear whether the 25% of subjects who did not normalise their IKDC score had specific symptoms, such as knee locking, and therefore represented a select subgroup of people who were unlikely to improve with conservative measures. It must be acknowledged that 75% of patients still improved to be normal or near-normal with regards to their IKDC scores in the nonoperative group, therefore avoiding APM.

## Meniscal repair

Whilst APM is one of the most commonly performed orthopaedic procedures [19], meniscal repair is the treatment of choice for the young athlete with an acute meniscal tear [20]. Meniscal repair is most commonly performed in younger populations aged under 35 years [10,21-27] and aims to preserve the entire meniscus, without excision. The rationale for meniscal repair is that by preserving the structure of the meniscus, function is maintained and long-term changes in other joint structures will be ameliorated.

The indications for performing a meniscal repair include: location (peripheral tears are located in a more vascular area and therefore heal better), morphology (shorter 2 cm tears and vertical longitudinal tears are more amenable to repair versus longer and degenerative horizontal tears) and chronicity (acute tears more amenable to repair). These conditions are rarely met in older patients, where degenerative meniscal tears are more apparent. Meniscal repair is thus performed exclusively in younger patients. Older patients are more likely to have degenerative tears that are not amenable to repair [28,29].

Currently, there are three arthroscopic techniques for meniscal repair in common use: the outside-in suture, the inside-out technique [30,31], and the all-inside technique that uses biodegradable products [32]. The outside-in technique uses spinal needles and sutures passed from outside to inside of the joint under arthroscopic observation. Tears in the anterior or body of the medial or lateral meniscus are easily accessed with the outside-in technique. For far posterior tears, which are harder to access, the inside-out or all-inside techniques are preferred [30].

## Evidence for meniscal repair

RCTs examining meniscal repairs have primarily focused on the differences in surgical methods, including failure and healing rates, as well as re-tear rates. In contrast, cohort studies have examined pain, function and radiographic knee OA as outcomes.

### Nonrandomised studies

Two cohort studies have compared meniscal repair to APM [25,27]. Melton and colleagues compared people requiring ACL reconstruction with the additional intervention of either inside-out suture repair (*n* = 35) or APM (*n* = 27) [27]. A control group requiring ACL reconstruction with intact menisci was also included (*n* = 40). The mean age of the patients in all treatment arms was less than 30 years. This study demonstrated that over a median follow-up of 10 years, the IKDC score for patients with meniscal repair was better than for those who underwent APM [27]. In another cohort study examining young adults, although it was shown that individuals who had APM returned to professional and sporting activities earlier than people with meniscal tear, there was less knee OA in the meniscal repair group at 7 year follow-up [25]. Similarly, a slightly larger cohort study of young athletes demonstrated that OA was less common in people who had previously had meniscal repair compared with those people who underwent APM [26]. In a follow-up case series of people who had longitudinal meniscal repairs with concurrent ACL repair when aged 20 years or younger, there appeared to be a chondroprotective effect from surgery, with a low rate of radiographic knee OA at a minimum of 10 years postoperatively [33]. Although there was a lack of a control group, the limited data may support the use of meniscal repair in select cases.

### Randomised controlled trials

Although five RCTs have examined the efficacy of meniscal repair (see Table 2), four of these studies have compared different surgical interventions, rather than comparing meniscal repair with other treatments, such as physical therapy. Nevertheless, regardless of the operative approach, symptom improvement occurred in most instances of meniscal repair, although superiority of one meniscal repair technique over another was generally not demonstrable.

**Table 2:** Evidence for meniscal repair

**Study**	**Study description**	**Group 1**	**Group 2**	**Group 3**	**Group 4**	**Outcome measure**	**Results**	**Conclusion**
**Randomised control trials**								
Jarvela and colleagues, 2010 [21]	Degenerative meniscal tear or knee OA excluded; 2-year follow-up	Screws, *n* = 21; mean age 30 years (±9 years)	Arrows, *n* = 21; mean age 32 years (±9 years)	N/A	N/A	Surgical failure. Structure	No between-group differences for surgical failure rate (*P* = 0.242). More chondral damage with arrows (*P* = 0.018)	Similar surgical outcomes. Arrows caused more chondral damage
Bryant and colleagues, 2007 [22]	Vertical meniscal tears only; 28-month follow-up	Sutures, *n* = 49; mean age 25.7 years (±9 years)	Arrows, *n* = 51; mean age 25.1 years (±8 years)	N/A	N/A	Re-tear rate. Symptoms and quality of life	No significant between-group differences for re-tear rate. No significant between-group differences for QOL or WOMET scores	No difference between the two different repair methods
Hantes and colleagues, 2006 [23]	Those with knee OA at arthroscopy excluded; 23-month follow-up	Group A: Outside-in, *n* = 17 (14 medial meniscus); mean age 28.5 years	Group B: Inside-out, *n* = 20 (17 medial meniscus); mean age 28 years	Group C: All-inside, *n* = 20 (17 medial meniscus); mean age 25 years	N/A	Operative time and healing rate	Healing rate in group C inferior to groups A and B. Group B was quickest procedure	Inside-out technique superior to other two as high rate of healing without prolonged operation time
Beidert, 2000 [10]	Painful intrasubstance medial meniscal tear; mean age 30.4 years (range 16 to 50 years); 26.5-month follow-up	Suture repair, *n* = 10	PT and NSAIDs, *n* = 12	Minimal resection, fibrin clot, suture repair, *n* = 7	APM, *n* = 11	Symptoms	Normal/near normal IKDC. Group 1, 75%; Group 2, 90%; Group 3, 43%; Group 4, 100%	Intra-substance (degenerative) meniscal tears were shown to be best treated by APM. Meniscal repair might give better medium-term to long-term results
Albrecht-Olsen and colleagues, 1999 [24]	Those with OA at arthroscopy excluded; 3-month to 4-month follow-up	Inside-out sutures, *n* = 32 (21 medial); median age 25.5 years (range 18 to 40 years)	All-inside meniscal arrows, *n* = 33 (21 medial); median age 26.5 years (range 18 to 37 years)	N/A	N/A	Healing rates	No between-group differences for healing (*P* = 0.11). No between-group differences in subgroup analyses, dependent on ACL reconstructed or ACL insufficient knees	Similar outcome with two meniscal repair procedures
**Cohort studies**								
Melton and colleagues, 2011 [27]	ACL lesions without degenerative changes; median 10-year follow-up; mean age 28 years (range 20 to 53 years)	Inside-out repair, *n* = 35 (32 medial); mean age 28 years	APM, *n* = 40; mean age 27 years	Intact menisci, *n* = 40; mean age 27	N/A	Symptoms	Mean IKDC significantly higher in meniscal repair group compared with menisectomy group	Improved functional scores achieved in people with ACL reconstruction and meniscal repair compared with ACL reconstruction and menisectomy
Stein and colleagues, 2010 [26]	Traumatic meniscal tear; mid-term follow-up at 3.4 years (*n* = 35); long-term follow-up at 8.8 years (*n* = 46)	Meniscal repair, *n* = 42; mean age 31.2	APM, *n* = 39; mean age 30.4	N/A	N/A	Structure and function	Significantly less progression of OA0 (*P* = 0.005); greater preinjury activity level (*P* = 0.001) and greater sporting activity among athletes (*P* = 0.001) in people treated with meniscal repair	Meniscal repair associated with better outcomes than APM
Sommerlath, 1991 [25]	Baseline symptoms not reported; knee OA excluded; 7-year follow-up	Open suture meniscal repair, *n* = 34; mean age 27 years	APM, *n* = 26; mean age 27 years	N/A	N/A	Symptoms. Structure	In meniscal repair group, significantly: higher LKS scores; less OA; longer return to professional activities	Reduced OA in meniscal repair group despite longer return to work than people receiving APM

ACL, anterior cruciate ligament; APM, arthroscopic partial menisectomy; N/A, not available; NSAID, nonsteroidal anti-inflammatory drug; OA, osteoarthritis; PT, physical therapy. Western Ontario Meniscal Evaluation Tool (WOMET) is a disease-specific quality-of-life measurement tool for patients with meniscal lesions looking at symptoms (pain, giving way, swelling, stiffness, numbness, loss of motion), sports/recreation/lifestyle/work and emotion. Quality-of-life (QOL) outcome measure consists of 32 items that address each of five separate quality-of-life domains: symptoms and physical complaints, work-related concerns, recreational activities and sports participation, life-style, and social and emotional concerns. Lysholm knee scoring (LKS) scale for knee ligament injuries including pain, swelling, locking, limping, stair climbing, support and squatting. International Knee Documentation Committee score (IKDC) is a score to evaluate knee ligament injuries including three domains of symptoms (pain, locking, catching, swelling, stiffness), sports and daily activities and current knee function (compared with old knee function).

To our knowledge, only one small study examining young adults has compared surgical interventions with conservative management (nonsteroidal anti-inflammatory drugs and physical therapy) [10]. This study compared four different treatment arms and found the following improvement at 26.5-month follow-up, as determined by clinical examination, IKDC score, radiographs and magnetic resonance imaging: conservative therapy (75%), arthroscopic suture repair with access channels (90%), arthroscopic minimal central resection, intrameniscal fibrin clot and suture repair (43%), and APM (100%). Such results demonstrated that APM and suture repair are superior treatments compared with conservative therapy in young adults over the short term, although in this small study there was no statistical difference between APM and meniscal repair groups.

## Conservative therapies

### Physical therapy

As discussed earlier, several RCTs have compared the effect of physical therapy or APM in people with a meniscal tear (see Table 3) [14-17]. No RCT examining older adults has demonstrated a significant difference in the symptoms experienced after completion of a physical therapy programme compared with APM. A smaller RCT, however, did show greater symptom improvement in younger people after APM than physical therapy, although 75% of people with physical therapy still had significant improvement in their knee pain [10].

**Table 3:** Evidence for conservative therapy

**Study**	**Study description**	**Group 1**	**Group 2**	**Group 3**	**Group 4**	**Outcome measure**	**Results**	**Conclusion**
**Physical therapy**								
Yim and colleagues, 2013 [16]	Degenerative horizontal tear of posterior horn of medial meniscus on MRI; mean age 53.8 years (range 43 to 62 years); 2-year follow-up	APM, *n* = 50	Strengthening exercises, *n* = 52	N/A	N/A	Symptoms	Both groups reported an improvement in knee pain, function and a high level of treatment satisfaction using VAS, LKS, Tegner activity scale, patient subjective knee pain and satisfaction. No significant between-group differences	APM not superior to strengthening exercises in terms of improved knee pain, function or treatment satisfaction
Katz and colleagues, 2013 [15]	Symptomatic meniscal tear; age ≥45 years; 6-month and 12-month follow-up	APM and postoperative PT, *n* = 161; mean age 59.9 ± 7.9 years	PT alone, *n* = 169; mean age 57.8 ± 6.8 years	N/A	N/A	Symptoms	WOMAC at 6 and 12 months: improvement in both groups but no between-group differences; 30% crossover from PT alone within first 6 months	PT non-inferior to APM + PT for pain reduction
Herrlin and colleagues, 2013 [17]	Symptomatic medial meniscal tear and radiographic OA; 24-month and 60-month follow-up	APM followed by exercise therapy for 2 months, *n* = 47; median age 54 years	Exercise therapy alone, *n* = 49; median age 56 years	N/A	N/A	Symptoms	Clinical improvement from baseline to the follow-up in both groups on all subscales of KOOS, LKS and VAS (*P* <0.0001). One third of exercise-alone patients that failed to respond had a benefit from then having APM	Exercise alone non-inferior to APM + exercise
Herrlin and colleagues, 2007 [14]	Knee pain and underlying OA with medial meniscal tear; mean age 56 years; 8-week and 6-month follow-up	APM and supervised exercise, *n* = 47	Supervised exercise alone, *n* = 43	N/A	N/A	Symptoms	Both groups reported decreased knee pain, improved function and high satisfaction. No between-group differences	Exercise alone non-inferior to APM + exercise
Beidert, 2000 [10]	Painful intrasubstance medial meniscal tear; mean age 30.4 years (range 16 to 50 years); 26.5-month follow-up	APM, *n* = 11	Suture repair, *n* = 10	Minimal resection, fibrin clot, suture repair, *n* = 7	PT and NSAIDs, *n* = 12	Symptoms	Normal/near-normal IKDC. Group 4, 75%; Group 2, 90%; Group 3, 43%; Group 1, 100%	APM superior to conservative therapy
**Weight loss**								
Teichtahl and colleagues, 2013 [34]	No previous diagnosis of knee OA; recruited from weight-loss clinics; mean age 45.7 years; 2.4-year follow-up	Medial meniscal tear on MRI, *n* = 36; mean age 51.0 ± 7.5 years	No medial meniscal tear on MRI, *n* = 161; mean age 45.8 ± 8.9 years	N/A	N/A	Structure. Symptoms	In people with medial meniscal tears: every 1% change in weight associated with change in medial tibial cartilage volume (95% CI 0.1 to 0.3%, *P* <0.001) and change in WOMAC knee pain (95% CI 2.1 to 21.1, *P* = 0.02)	Weight loss associated with reduced cartilage loss and improved pain only in people with medial meniscal tears. Weight gain increased cartilage loss and knee pain

APM, arthroscopic partial menisectomy; CI, confidence interval; MRI, magnetic resonance imaging; N/A, not available; NSAID, nonsteroidal anti-inflammatory drug; OA, osteoarthritis; PT, phsycal therapy. Western Ontario and McMaster Universities Arthritis Index (WOMAC) evaluates the condition of patients with osteoarthritis of the knee and hip, including pain, stiffness, and physical functioning of the joints. Knee Injury and Osteoarthritis Outcome Score (KOOS) evaluates short-term and long-term patient-related outcomes following injury including pain, other symptoms such as catching/locking/swelling, activities of daily living, sport and recreation function, and knee-related quality of life. Lysholm knee scoring (LKS) scale for knee ligament injuries including pain, swelling, locking, limping, stair climbing, support and squatting. Visual analogue scale (VAS) is a subjective measurement of pain consisting of a line 10 cm long where on one end is ‘no pain’ and on the other is the ‘worst pain imaginable’. International Knee Documentation Committee score (IKDC) is a score to evaluate knee ligament injuries including three domains of symptoms (pain, locking, catching, swelling, stiffness), sports and daily activities and current knee function (compared with old knee function).

### Weight loss

A recent cohort study examined the effects of weight change on knee pain in participants with and without meniscal tears (Table 3) [34]. Two hundred and fifty participants (mean age 46.7 years) with no history of knee OA were recruited from the community, and the outcome measures were change in cartilage volume and Western Ontario and McMaster Universities Arthritic Index knee pain measured over approximately 2.4 years. Methods for weight loss included either surgical (laparoscopic adjustable gastric banding) or caloric restriction. Results showed that small increases in weight expedited cartilage volume loss and increased knee pain among people with medial meniscal tears only. Conversely, as little as 1% loss of weight was associated with a reduction in the rate of cartilage volume loss and an improvement in knee pain in people with, but not without, medial meniscal tears. This study suggested that in adults with medial meniscal tears, attention to weight is an important factor in the conservative management of meniscal tears.

## Meniscal tears – the treatment conundrum

Although there is a paucity of evidence examining the efficacy of treatments available for meniscal tears, there are some commonalities emerging from both RCTs and observational studies.

Overwhelming evidence has demonstrated the poor long-term outcomes from total menisectomy, and this procedure should be considered a procedure of yesteryear [4-6]. All APM, meniscal repair and physical therapy have been shown to be associated with symptom improvement, although there are conflicting data regarding the comparative superiority of the surgical versus the conservative approach. While one RCT demonstrated that APM was superior to conservative measures, the between-group comparison was limited by a small number of subjects (*n* = 23) who were young (mean age 30.4 years) [10]. The other four larger RCTs have examined older subjects and have failed to demonstrate superiority of APM over conservative measures [14-17]. This discrepancy between studies may be attributable to the different ages of the cohort.

With the passage of time, OA changes within the joint accumulate and age may be a surrogate for cumulative joint damage. In this instance, an operative approach in older persons may not yield as much symptomatic benefit, because other abnormalities in joint structures may contribute to the knee pain and reduced function which prompted medical attention to be initially sought. In people with radiographic knee OA, 63% of people with knee pain have co-existing meniscal tears while 60% of people without knee pain also had a meniscal tear [1]. This begs the question of whether the degenerative meniscal tear is a contributor to the joint symptoms or is simply a marker of the OA process. Age also appears to be a major determinant of the surgical approach as meniscal repair has been performed exclusively in younger populations whereby OA changes have been an exclusion criterion. This is presumably because whereas middle-aged and older adult meniscal tears are degenerative, younger patients have menisci that are more amenable to repair. Nevertheless, there may be rare circumstances where meniscal repair may be indicated in the older adult, provided there is good quality tissue, an acute tear and appropriate vascularity.

In the small RCT performed in young adults that demonstrated the superiority of APM over conservative measures, 75% of subjects undergoing treatment with nonsteroidal anti-inflammatory drugs and physical therapy still had improvement to normal or near-normal IKDC scores. The added economical and surgical risks associated with APM may not justify this approach as first-line therapy. Indeed, an emerging theme in most of the RCTs is that approximately one-third of subjects did not have an adequate response to conservative measures. APM may therefore only be indicated in people who do not respond to conservative therapies. Whether or not this can be generalised to people who have mechanical symptoms, such as knee locking, is unclear since studies have not specified which knee symptoms were present. Stratifying outcomes according to the indication of the surgery being either knee pain or mechanical symptoms may help to better understand which individuals may benefit from a surgical approach. Perhaps surgery is beneficial when mechanical symptoms predominate. Likewise, the location and type of meniscal tear may be an important and underappreciated determinant of treatment response, and future efforts should focus on examining this possibility.

Finally, studies have predominantly defined outcome on the basis of symptoms. There is no clear evidence as to whether the risk of developing knee OA, or showing increased progression of knee OA, is affected by surgical intervention comprising either APM or meniscal repair. Determining whether these procedures influence structural progression (for example, alter the rate of cartilage loss, metaphyseal bone expansion, incident or progressing bone marrow lesions, and so forth) will be important in helping to understand whether such procedures impart deleterious structural changes.

## Conclusions

Surgical approaches to the treatment of meniscal pathology can be broadly categorised as menisectomy or repair. Clearly the major distinguishing factor determining the choice of operative approach is age, whereby meniscal repair is performed exclusively on younger populations while older populations undergo menisectomy procedures. Nevertheless, middle-aged to older adults with meniscal tears often have coexisting degenerative changes in other knee joint structures. In such populations, APM may be treating the meniscus tear in isolation and not addressing the degenerative whole-organ disease of knee OA. Thus far, there is no convincing evidence that demonstrates the superiority of operative approaches to conservative measures for the treatment of people aged approximately 45 years or older with knee pain and meniscal tears. The available data would support a trial of conservative therapy as first-line treatment of meniscal tears in older adults, before consideration of APM. Studies have not, however, stratified populations according to mechanical symptoms, such as knee locking, and it may be that operative interventions are required to derive functional benefits in such subgroups. In younger individuals, meniscal repair is possible, although there is a paucity of data to explore whether this provides greater symptom benefit than conservative measures.

## Abbreviations

ACL: Anterior cruciate ligament; APM: Arthroscopic partial menisectomy; IKDC: International Knee Documentation Committee; OA: Osteoarthritis; RCT: Randomised controlled trial.

## Competing interests

The authors declare that they have no competing interests.
